# Overcoming the Biological Contamination in Microalgae and Cyanobacteria Mass Cultivations for Photosynthetic Biofuel Production

**DOI:** 10.3390/molecules25225220

**Published:** 2020-11-10

**Authors:** Zhi Zhu, Jihong Jiang, Yun Fa

**Affiliations:** 1 The Key Laboratory of Biotechnology for Medicinal Plants of Jiangsu Province and School of Life Science, Jiangsu Normal University, Xuzhou 221116, China; zhuzhi@jsnu.edu.cn; 2 CAS Key Laboratory of Bio-Based Materials, Qingdao Institute of Bioenergy and Bioprocess Technology, Chinese Academy of Sciences, Qingdao 266101, China

**Keywords:** microalgae, cyanobacteria, biofuel, biological contamination, controlling strategy

## Abstract

Microalgae and cyanobacteria have shown significant potential for the development of the next biofuels innovation because of their own characteristics as photosynthetic microorganisms. However, it is confronted with a lot of severe challenges on the economic scaling-up of the microalgae- and cyanobacteria-based biofuels production. One of these major challenges is the lack of a reliable preventing and controlling culture system of biological contamination, which can attack the cell growth or product accumulation causing crashing effects. To increase the commercial viability of microalgae- and cyanobacteria-based biofuels production, overcoming the biological contaminations should be at the top of the priority list. Here, we highlight the importance of two categories of biological contaminations and their controlling strategies in the mass cultivations of microalgae and cyanobacteria, and outline the directions that should be exploited in the future.

## 1. Introduction

The worldwide increasing environmental pollution issues associated with the use of fossil fuels as well as their expected scarcity in the near future have triggered the need for exploring new alternative fuels produced from clean, efficient, and sustainable production processes, such as biofuels [1,2,3,4]. Biofuels, composed of, derived from or produced by microbial cell factories from biological resources, are presently being recognized as drop-in fuels, and the usage of biofuels, such as biodiesel, bioethanol, and jet fuel, has increased immensely, especially in the transport industry where electrification is not available [5,6].

Based on the source of feedstocks and bioprocess technologies, four generations of biofuels have been developed. First-generation biofuel is derived from grain crops and oleaginous plants [7]. Second-generation biofuel is intended to use nonedible lignocellulosic materials [8]. However, these two generations have major disadvantages of “competing with human for food crops” or “competing with crop plants for fertile land”. Third and fourth generations produce biofuels based on photosynthetic microbes like eukaryotic microalgae and/or prokaryotic cyanobacteria, which do not compete for food sources and arable land [9]. Third-generation biofuel mainly involves improvement of biomass production to increase lipid accumulation while the fourth generation aims to use tools and strategies of systems biology and genetic engineering to enhance the direct production of algae biofuels [10,11,12].

The utilization of microalgae and cyanobacteria could provide numerous advantages in common, such as the higher growth rate and photosynthetic efficiency compared to plants, the ability to thrive in areas that cannot support agriculture, and the high degree of environmental tolerance [13,14]. Separately, microalgae provide a completely biodegradable source and sulphur-free lipid for biofuels [15]. Development in bioprocess engineering and increased knowledge of algal physiology have paved the way for their use in biofuel applications [16]. Cyanobacteria have attracted increasing attention nowadays as a great potential photosynthetic platform with a simple background and convenient genetic manipulations [17,18]. Modification of the natural metabolism network or introduction of heterologous metabolic pathways into the metabolism of cyanobacteria has allowed photosynthetic production of a wide range of small-molecule biofuels, such as ethanol, butanol, and isoprene [5,19,20,21,22,23,24].

Both microalgae and cyanobacteria biomanufacturing have proved to be a promising solution for sustainable production of biofuels, so that a large number of research and development programs in this field are in progress worldwide [25,26]. However, commercialized applications of microalgal- and cyanobacterial-based photosynthetic biofuels are far from economically feasible [27]. Besides the genetic engineering strategies [28,29], the cultivation process optimizations [30,31], and advanced photobioreactor designs for scale-up production [32,33,34], another pressing challenge hindering the scaling-up processes of microalgae or cyanobacteria is the inescapable biological contamination [35,36,37]. As for large-scale mass cultivations, cellular biomass accumulation of microalgae or cyanobacteria is usually inhibited or devastated by different types of biological contaminants at different growth stages while decreased biomass of microalgae or cyanobacteria usually result in the corresponding decrease of the lipid productivity [38]. In the case of direct transformation of “algae-to-biofuels” by photosynthetic chassis cells, production of the target products could influence and change the overall environment conditions, and further lead to specific contamination patterns. Therefore, it is increasingly recognized that we need a better understanding of the biological contaminants and their contamination patterns in the mass cultivations by microalgae and cyanobacteria for biofuels productions. More significantly, devising prompt solutions for controlling biological contamination has become a crucial problem that urgently needs to be addressed.

**Figure 1:**
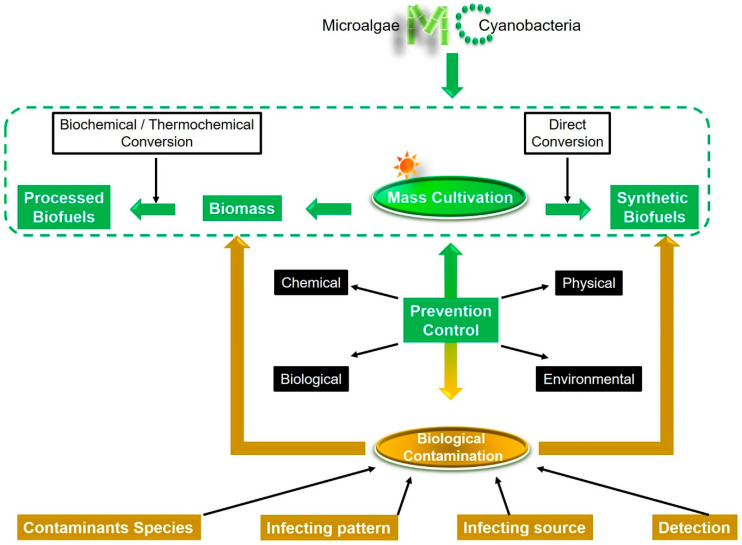
Prevention and control of the biological contamination should be at the top of the priority list for increasing the commercial viability of microalgae- and cyanobacteria-based biofuel production.

This review aims at updating progress on the biological contaminations and the controls in the mass cultures with microalgae and cyanobacteria for biofuels production. We emphasize here the significance of preventing and controlling both biomass-related contamination and synthetic product-related contamination emerging in the direct algae-to-biofuels production should be exploited in the future (Figure 1).

## 2. Major Biological Contaminations and the Infection Patterns

The biological contamination is a widespread problem during mass cultivations of microalgae and cyanobacteria because the contaminants will inevitably go into the culture by water or air. Depending on the objectives, these biological contaminants can be reducible to two groups, one of which can significantly constrain or destroy the cell growth of the microalgae or cyanobacteria strain, while the other can significantly inhibit or consume the products produced or secreted by microalgae or cyanobacteria.

### 2.1. Cell Growth-Affecting Contaminations

At present, in general, cell growth-affecting biological contaminants that emerged in mass cultivations of microalgae and cyanobacteria are summed up in four forms: zooplankton, microorganisms, including prokaryote bacteria, eukaryotic fungi, and eukaryotic protozoa; other algae; and virus [39,40,41]. Wang summarized the ubiquitous diverse contamination mechanism according to each form of the biological contaminants, i.e., zooplankton employed a positive or negative feeding mechanism, phytoplankton-lytic bacteria employed direct or indirect attack, harmful algae employed direct cell contact, nutrient competition or allelopathy mechanism, and virus employed a host-specific infection mechanism [40]. Molina considered the biological contamination mechanisms were related to the existing natural interactions, such as mutualism, commensalism, and parasitism [41]. These enormous types of contaminants in the cultures are deleterious for the biomass accumulation and the lipid production, and frequently lead to a loss of the entire biofuel production (Table 1). In fact, with respect to the enhancement of lipid productivity, a higher growth rate is more important than the higher lipid content of the microalgal or cyanobacterial cells [38]. Currently, contaminations caused by grazing of zooplankton are still the most serious examples, of which, rotifers like *Brachionus calyciflorus* and *Brachionus plicatilis* were considered the most common but destructive grazer [42]. Fischer reported that rotifer *B. calyciflorus* is able to consume more than 500 cells of *Chlamydomonas reinhardtii* per hour per rotifer [43]. Lubzens’s study showed that rotifer *B. plicatilis* can eat about 4800 cells of *Nanochloropsis* sp. per hour per rotifer, thus resulting in pond crash in a few hours [44,45,46].

### 2.2. Products Accumulation-Affecting Contaminations

With the development of systematic metabolic engineering and synthetic biology, engineered microalgae and cyanobacteria, especially prokaryote cyanobacteria, have attracted attention as a “biosolar cell factory” for the direct conversion of carbon dioxide into biofuel products [27]. Experimental pilots for some biofuel compounds have been evaluated for the economic feasibility. However, besides the risk of cell growth-affecting contaminations during the photosynthetic production, biological contaminants can also significantly destroy the direct conversion processes of biofuel compounds by engineered cyanobacteria or microalgae. In the efforts to scale up the photosynthetic ethanol production with a previously developed cyanobacterium *Synechocystis* strain in our work [24,47], biological contamination was also the main threat, and the synthetic product ethanol became the subverting object instead of the cyanobacteria cells. Ethanol accumulation in the outdoor non-sterilized cultivation system was completely consumed and devastated by at least one specific invading contaminant microorganism, which was identified by 16S RNA as *Pannonibacter phragmitetus*. Physiological analysis proved that this contaminant *P. phragmitetus* really could grow in BG 11 medium with ethanol as the sole carbon source, and the consumption rate of ethanol reached 0.37 g per liter per day, 68% higher than the ethanol productivity of current engineered cyanobacteria [37]. There have been few synthetic product-related contamination cases reported so far; however, the seriousness of the synthetic product affecting contaminations cannot be overstated. Along with the wide applications of a “biosolar cell factory” in biofuel mass productions, synthetic product-related contaminations should primarily be given close attention during scaling-up processes.

Microalgal and cyanobacterial biomass-based biofuels belong to the third generation of biofuels, while direct converted biofuels based on microalgal and cyanobacterial cell transformation platforms are proposed as the fourth generation of biofuels; both third and fourth generations are considered as a viable alternative energy source to replace or supplement fossil fuels [48]. All the above-mentioned cell growth-affecting contaminants and product accumulation-affecting contaminants exhibit productivity restriction of biofuel productions based on microalgae and cyanobacteria (Table 1), especially in the large-scale open system. To accomplish the biorefinery of these biofuels on a commercial scale, it is of key importance to make sure the microalgal or cyanobacterial cells grow normally in the first place. For this reason, it is essential to obtain a close understanding of the cell growth-affecting biological contaminants emerging in the mass culture, which can highly jeopardize the biomass accumulation of microalgae or cyanobacteria. Along with the recent progress of metabolic engineering and systematic biology, prokaryotic cyanobacteria and some eukaryotic microalgae have gained fascinating attention as promising biosolar cell factories for biofuels production. Consequently, specific biological contaminations have been emerging in the scaling-up processes of this technology. Thus, it is of paramount importance to accelerate the understanding of the product accumulation-affecting biocontaminants, which do not damage the cell growth of microalgae or cyanobacteria; however, they still have the ability to destroy the entire algae-to-biofuels production.

**Table 1:** Representative of biological contaminants in the cultures of microalgae and cyanobacteria.

Categories of Biological Contaminants	Species of Biological Contaminants	Microalgae/Cyanobacteria Host	Culture System	Impact of Contamination	Reference
Zooplankton	Rotifer *Brachionus calyciflorus*	*Chlorella kessleri*	Laboratory condition	Pond crash within days of infection	[44,46]
	*Amoeboaphelidium protococcarum*	*Scenedesmus dimorphus*	400 L outdoor ponds	Rapid event with devastating consequences for the algal population	[49]
	Ciliates	*Dunaliella salina*	Laboratory condition	Clarified the algal culture within 2 days	[50]
Fungi	Chytrid (phylum *Blastocladiomycota*)	*Haematococcus pluvialis*	Laboratory condition	Caused epidemics resulting in damage to the cultures	[51]
Other Algae	Golden algae (*Poterioochromonas* sp.)	*Synechocystis* sp. PCC 6803	1 L Pyrex Roux-type photobioreactor	Killing effect on the culture	[52]
Virus	*Heterosigma akashiwo* virus (HaV)	*Heterosigma akashiwo*	Laboratory condition	Algal culture became transparent within 33 h after inoculation	[53]
Products-Consuming Bacteria	*Pannonibacter phragmitetus*	*Synechocystis* sp. PCC 6803	6 L hanging membrane photobioreactor	Accumulation of the target product ethanol was exhausted	[37]

## 3. Infecting Sources of Biological Contaminants

In order to prevent and control the biological contamination, besides the infection patterns, we should also explore the infecting sources of the biological contaminants. Whatever the materials or the structures of the photobioreactors used in mass cultivations of microalgae and cyanobacteria, biological contaminants are inevitable by large volumes of input medium, gas exchange, and the blind angles of photobioreactors. The infecting sources of biological contaminations are summarized as “AAA” as follows.

### 3.1. Aquatic Pollution

Existing commercial mass culture systems, including open pond systems and closed photobioreactors, range in volume from <1 to >10^7^ m^3^ [54,55]. According to Tu’s study, the water consumption rate at the growth stage of microalgae ranged from 165 to 2000 gallon/gallon biodiesel [56]. Apparently, the mass cultivation of microalgae and cyanobacteria relies on a large body of water. Therefore, they cannot be sterilized by the wet heat sterilization method, which is usually used in conventional microbial fermentation processes. Though the medium can be disinfected by bleaching or filtration before mass cultivations of microalgae and cyanobacteria, it usually lasts for only the growth lag phase and there is still the chance that a certain amount of contaminants may remain because of the low chlorine concentration, short treatment time, or insufficient filtration [40].

### 3.2. Air Pollution

The exposure of culture to air is helpful for atmospheric CO_2_ input and removal of excess dissolved oxygen when open ponds are used in mass culture of microalgae and cyanobacteria [57]. However, this open culture mode creates more opportunities, letting the biological contaminant from the air invade the cultivation system, and severely restricting the species of microalgae and cyanobacteria that can be applied in outdoor mass cultivation. Even culturing in the closed photobioreactors, cultivation of microalgae and cyanobacteria and agitation of the bulk volumes of cultures need the continuous supplement of air or CO_2_-air gas mixture. The more gas exchange during the cultivation, the greater the chance of biological contamination occurring.

### 3.3. Blind Angles of Photobioreactor

Photobioreactors with different materials and structures have different blind angles, such as the immobilized gas distributor of the membrane photobioreactors, the pipe connection of the tubular photobioreactors, and the sampling system of all kinds of bioreactors. The nutrient, salt, or the microalgae and cyanobacteria cells that remain in these blind places not only cause the contamination of biological pollutants in the next cultivation but also accelerate the bioreactors’ corrosion. In addition, the continuous cultivation mode is always applied in the practical mass culture, making the risk lying in the contamination associated with the residual contaminants attached in the blind angles even higher.

The specific characteristics of microalgae- and cyanobacterial-based biofuels create the significant advantages as mentioned before, which, however, is a two-edged sword, and the current economically potential mass culture system has led to biological contamination being inevitable. Even the solid culture mode, like attached cultivation [58], supplement of Aquatic nutrient solution, exchange of CO_2_ mixed air, and the contaminants hidden at the blind angles, is also inescapable during the mass culture. Therefore, early detection and early treatment can significantly increase the chance of success for microalgae- and cyanobacterial-based biofuels bulk productions.

## 4. Detection of Biological Contaminants

Detection of biological contaminants during the mass cultivation of microalgae and cyanobacteria is an important step before adopting possible measures for the culture system. Conventional morphologic observation methods, such as staining and microscopy, can be applied as an early and routine detection method [59]. The way we initially detected the contaminant in the cultivation of engineered cyanobacteria was by comparing the microscopic images at different product accumulating stages during the culture process. Gerphagnon applied a modified staining method, which combined chitinous fluorochrome and nucleic acid stain coupled with epifluorescence microscopy for characterization of the chytrid fecundity of cyanobacterium Anabaena macrospora [60]. Considering the large-scale industrial application, automated detection systems would be ideal for the early warning analysis. An automated flow cytometry and microscopy detection system named FlowCAM was constructed for the characterization of marine microplankton in the size range of 20–200 μM [61]. A modified FlowCAM system has been developed for automated monitoring in microalgae cultivation, where the in situ microscope obtained on-line data of cell number, size, and morphology during the culture of *Chlorella vulgaris* over 20 days, and the biomass data was totally in agreement with the off-line measurement [62]. Recently, modern molecular-based methods associated with new-generation sequencing technologies and quantitative PCR technology are becoming great sensitive and informative methods for the purposes of identification and characterization of biological contaminants [59]. Furthermore, specific metabolites can also be the contaminating reporters, such as decreasing ethanol, which is consumed as a carbon source by the biological contaminant in our case of exploring photosynthetic ethanol production with an engineered *Synechocystis* strain [37].

Compared to the synthetic product-affecting contaminations, it is often easier to observe the phenomenon of cell growth-affecting biological contaminations, i.e., cultures gradually becoming yellow or clear. For synthetic product-affecting contaminations, not only might biological contaminants have no impact on the growth of microalgae or cyanobacteria but they also improve the photosynthetic growth. Therefore, a simple and fast detection method like an automated flow cytometry system could provide us an ideal early warning of biological contamination for cell growth-affecting biological contaminants. The detection method associated with the accumulation of the target product might be feasible for monitoring the mass cultivation process during the direct algae-to-biofuels production. In the practical algae-to-biofuels production, cell and target product monitoring detection methods should stand by before mass cultures. All the detected information and identified characterization of biological contaminants could further provide us the specific features for designing the specific control strategies.

## 5. Strategies for Controlling Biological Contamination

The objective of studying the biological contamination types and the corresponding infecting patterns, infecting sources, as well as the detection methods of bio-contaminants is to propose or develop reliable solutions to greatly control the biological contamination level and still prevent transmission, thus healing the entire photosynthetic biofuel productions during the mass cultivation of microalgae and cyanobacteria.

### 5.1. Chemical Control

Chemical control of microalgae and cyanobacteria cultivation systems usually serve as one feasible solution for controlling biological contaminants. As mentioned before, because of the large body water for biofuel production, hypochlorite or bleach are usually widely used to sterilize the culture medium before or during the culture processes of microalgae and cyanobacteria. Park’s results indicated that the dosage of sodium hypochlorite ranged from 0.45 to 0.6 mg Cl/L and enabled the chlorine concentration effective for inhibiting the grazing of rotifers while maintaining the growth of the microalgae *Chlorella kessleri* [44]. Conventional chemical pesticides are also extensively used in protecting microalgae and cyanobacteria cultivations from biological contaminations. Rotifers or ciliates can be effectively killed by organophosphorus or organonitrogen insecticides, such as trichlorphon, dimehypo, methyl parathion, and diazinon [63,64,65]. Surfactant Triton-N can be used in keeping the inoculum healthy as this surfactant can inhibit the growth of Scenedesmus in a short time [66]. Chemical reagents, such as copper salts, have been reported for use as a fungicide against chytrids, and the addition could accelerate the algae productivity because it is required as a trace element for algal growth during cultivation [67]. It seems that chemical control may be industrially available; however, it is worth noting that most chemical reagents could also inhibit the growth of aquatic organisms per se and reduce the concentration of phycocyanin [65]. In addition, the excessive applications of these synthetic chemical reagents have the potential hazards of the global environment and human health [42,68]. Therefore, screening of environmentally friendly biological agents that inhibit the multiplication of biological contaminants without damaging the cells of microalgae and cyanobacteria should be further investigated.

### 5.2. Biological Control

Applying specific pathogens to control biological contaminants, which break the limitation of conventional chemical methods and overcome the effect of toxicity toward the cell of microalgae or cyanobacteria, may be the ideal contamination controlling strategy in mass cultivation. The implementation of such biological control, however, is dependent on the specific selection to the contaminants. Such selection requires detailed information about the relationships between the contaminants and the microalgae or cyanobacteria strains, which need further investigation [59].

Compared with the chemical reagents and specific pathogens, plant-derived pesticides may be the preferred choices for controlling biological contaminants in mass culture systems of the microalgae- and cyanobacteria-based biofuel production. According to Huang’s acute and chronic tests, celangulin, matrine, and toosendanin are considered to be good potential rotifer-control botanical pesticides in microalgal mass culture [42]. In addition, rotifer-control effects of the synergistic celangulin/toosendanin combination for *Spirulina platensis* growth were further investigated; here, 0.003–0.006 mg/L of the botanical pesticide combination at a 1:9 ratio can inhibit rotifer regeneration within 3 days, and no influences on the *Spirulina* biomass and phycocyanin levels showed up [69]. Zhang also found that the celangulin/toosendanin combination at 1:9 could eliminate the breeding of *Brachionus plicatilis*, thus healing the photosynthetic performance of *nannochloropsis* cells against the contamination damage of rotifer [70]. Therefore, plant-derived pesticides exhibit excellent potential and viability for controlling biological contaminations in mass cultures of microalgae and cyanobacteria for biofuels productions.

### 5.3. Physical Control

Physical filtration and ultraviolet sterilization have been considered as effective and common methods for removal of biological contaminants, but only before the culture starts on the one hand. On the other hand, the removal efficiency of filtration is dependent on the cell size, which also limits the application in treating biological contaminants, such as rotifer eggs and copepod eggs. Ultrasound treatment has also been investigated for the effect on the survival of bacteria, phytoplankton, and zooplankton [71]. The effective dosage, again, is dependent on the cell size of the contaminants. The new application of pulsed electric fields in order to control contaminating rotifer in the cultivation of microalgae appears to be very suitable to carry out on an industrial scale, as it can selectively impact the rotifer but not the microalgae in cell concentration. Furthermore, it neither needs any chemical agent to the cultures nor any significant modification of the existing culture facilities [72].

### 5.4. Environmental Control

Microalgae and cyanobacteria are among the most primitive life forms on our planet, and they have the ability of adaptation to almost any of the most extreme habitats on Earth [73]. Therefore, lots of microalgae and cyanobacteria strains are tolerant to extreme environmental culture conditions, such as environmental pH, temperature, and light intensity. Adjusting these environmental conditions to a specific stress range at which the microalgal or cyanobacterial cell can survive while the biological contaminants cannot may be an ideal and environment friendly strategy for controlling the biological contamination level. The most successful paradigm of commercial cultivation of algae is spirulina with extreme high pH conditions and *Dunaliella* with extreme high-salinity conditions [74,75,76]. Touloupakis’ work also found that the cyanobacteria *Synechocystis* strain was alkaline tolerant, while the golden algae *Poterioochromonas* strain was sensitive to alkaline stress at pH 11; thus, a high-pH culture strategy was designed and adopted to selectively inhibit or even kill the golden algae [52]. In our scaling-up process of ethanol photosynthetic production with an engineered *Synechocystis* strain, infection of an ethanol-consuming biocontaminant gradually ceased and devastated ethanol accumulations, and we found that a high-pH strategy maintaining culture environmental pH in an alkaline condition (pH 10–11) could successfully inhibit the contamination level and recover the photosynthetic ethanol accumulations [37]. In addition, rising environmental pH conditions using bicarbonate integrated low concentration carbon dioxide as a carbon capture system not only achieves a high pH environment ideally but also increases the carbon capturing efficiency and can easily realize industrialized amplification. Light shock (sudden increase of the light intensity to 30,000 Lux), salinity shock (over 15% NaCl), and temperature shock (temperature could explain 90% of the variance in the copepods’ growth rate) could also influence the grazing relation between microalgae or cyanobacteria and the protozoa [77,78,79].

Biological contamination can occur in any stage of the mass cultivations for biofuel photosynthetic production in the form of chronic or acute infection. Four main categories of control approaches as shown in Table 2, and play important roles in controlling the contamination level. However, every treatment has its deficiency, and there is no single solution that can address all the contamination problems. Furthermore, biological contamination during the mass cultivation of microalgae and cyanobacteria is almost inevitable, but it is uncertain which kind of contamination would happen. To overcome the shortcomings of any single control strategy and the lack of contaminants’ information, the construction of a control system for each cultivation must be the combination of different strategies and based on the specific physiological features of the contaminants. Learning from our research experience of scaling up of “cyanobacteria-to-ethanol” production, environmental control might be the most efficient solution. Thus, for the photosynthetic biofuel production of microalgae and cyanobacteria, it is ideal to develop a set of controlling culture systems based on the environmental controlling strategies. Effective controlling of the contamination level, facilitating the substrate consumption or product production, as well as industrial application potential should be worthwhile goals for the contamination controlling culture system for the biofuel production of microalgae and cyanobacteria.

## 6. Concluding Remarks and Future Perspectives

Photosynthetic biorefinery engineering provides a promising solution for sustainable biofuels production. Microalgae and cyanobacteria are among the most promising photosynthetic platforms. Considering economic and operation feasibilities, microalgae and cyanobacteria-based biofuels must be performed at a large scale under open systems, which, however, means potential infection and proliferation of diverse biological contaminants. To promote the concept of photosynthetic biofuel in practice, intervention strategies against biological contaminations through the outdoor mass cultivation process must be overcome and economically feasible. Developing a reliable control system for the mass cultivation by combining the specific controlling strategies coupled with early detection, which can serve to not only prevent but also control function, may be a long-term plan. For further optimization of the mass cultivation processes of microalgae and cyanobacteria for biofuels production, more robust photosynthetic platforms with commercialization potential should be constructed, which could be expected by the development of more powerful evolutionary approaches and metabolic engineering, leading to economically competitive scaled photosynthetic biofuels production.

## Figures and Tables

**Table 2:** Four categories of solutions to control the biological contaminations.

Solution Type	Controlling Treatment	Reference
Chemical Control	Copper Salts Surfactant Triton-N Trichlorphon	[67] [66] [65]
Biological Control	Specific pathogen	[59]
	Celangulin/toosendanin(1:9)	[69,70,80]
Physical Control	Filtration Pulsed Electric Fields Sonication	[40,59] [72] [59]
Environmental Control	High pH High salinity Light shock High temperature	[52,74] [81] [77] [79]
